# A longitudinal study of semen quality among Chinese sperm donor candidates during the past 11 years

**DOI:** 10.1038/s41598-020-67707-x

**Published:** 2020-07-01

**Authors:** Junjie Liu, Yanpeng Dai, Yushan Li, Enwu Yuan, Quanxian Wang, Xingling Wang, Yichun Guan

**Affiliations:** 1 Henan Human Sperm Bank, The Third Affiliated Hospital of Zhengzhou University, No. 7 Front Kangfu Street, Er’qi District, Zhengzhou, 450052 China; 2 Department of Clinical Laboratory, The Third Affiliated Hospital of Zhengzhou University, No.7 Front Kangfu Street, Er’qi District, Zhengzhou, 450052 China; 3 Center for Reproductive Medicine, The Third Affiliated Hospital of Zhengzhou University, No.7 Front Kangfu Street, Er′qi District, Zhengzhou, 450052 China

**Keywords:** Environmental sciences, Health care, Urology

## Abstract

Studies suggest that semen quality is declining globally, however, the debate remains open due to the possible effects of ethnic and geographical differences. This study aimed to explore whether semen quality of sperm donor candidates has changed in Henan Province, China from 2009 to 2019. In this retrospective study, we included 23,936 sperm donor candidates who were recruited by the Henan Human Sperm Bank of China between 2009 and 2019. To minimize intra-individual bias, we included only the first ejaculate provided by each sperm donor candidate. The following parameters were measured: volume, sperm concentration, total sperm count, progressive motility, and total motility. After adjustment for age, body mass index (BMI), and sexual abstinence duration, we evaluated changes in main semen parameters over time using multiple linear regression analyses. The sperm concentration decreased from 62.0 million/mL in 2009 to 32.0 million/mL in 2019 (*P* < 0.001), with an average annual rate of 3.9%. The total sperm count decreased from 160.0 million in 2009 to 80.0 million in 2019 (*P* < 0.001), with an average annual rate of 4.2%. The progressive motility decreased from 54.0% in 2009 to 40.0% in 2019 (*P* < 0.001), with an average annual rate of 2.5%. The total motility decreased from 60.0% in 2009 to 46.0% in 2019 (*P* < 0.001), with an average annual rate of 1.9%. Our results indicated that semen quality among sperm donor candidates had decreased during the study period in Henan Province, China.

## Introduction

The semen analysis is the most important and the most widely used clinical laboratory test to evaluate male fertility potential. In 2010, the World Health Organization (WHO) criteria had reduced the reference interval of sperm concentration from 20 to 15 million/mL. Concerns about a global decline in semen quality have attracted the attention of researchers and the general public alike. There is an ongoing debate on whether semen quality is declining. In 1992, Carlsen et al^1.^ reviewed 61 studies and found a worldwide decline trend in semen volume and sperm concentration between 1938 and 1991. After that, several studies have reported a decrease in semen quality in Paris^2^, Tours^3^, Marseille^4^, São Paulo^5^, Shandong^6^, Changsha^7^, and South India^8^. In contrast, several other studies have reported no significant change in semen quality in Sydney^9^, Malmö^10^, Montevideo^11^, and Copenhagen^12^. These discrepancies could be explained by ethnic, geographical, lifestyle, environmental and between-center variations^13–16^.

To accurately evaluate changes in semen quality, ethnic, geographical, and between-center variations must be taken into account^15,16^. A single-center retrospective investigation of possible changes in semen quality, using the same equipment and based on a large population over a long period of time, has been recommended to minimize these confounding factors^17,18^. Based on this, laboratories must conduct their local studies over a certain period of time. Large and long-term population studies on time trends in semen quality have been reported in Hunan Province^19^ but not in Henan Province of China. This study aimed to analyze the main semen parameters of 23,936 sperm donor candidates from Henan Human Sperm Bank of China and to look at changes that have occurred during the past 11 years.

## Materials and methods

### Subjects

Men were encouraged to be volunteer sperm donors through newspaper advertisements in magazines, newspapers, news forums, social media and personal contact with clinic staff. In this retrospective study, we enrolled 23,936 sperm donor candidates who were recruited by the Henan Human Sperm Bank of China between 2009 and 2019. All the sperm donor candidates in our study were aged between 20 and 44 years with no selection for marital status or fertility. Most of the sperm donor candidates knew nothing about their fertility status during their first consultation. Potential sperm donors were screened strictly following the guidelines published by the Ministry of Health of China in 2003. Sperm donor candidates were recruited in this study by the following criteria: (1) 20–45 years old; (2) height of 168 cm or taller; (3) currently attending or have graduated from a junior college; (4) healthy. The following information was obtained from all participants: age, weight, and height. Body mass index (BMI) was calculated using objectively measured weight and height (kg/m^2^). All donor candidates were given an anonymous code number. Identifying information was known only to the staff of the Henan Human Sperm Bank. This study was approved by the ethics committee of the Third Affiliated Hospital of Zhengzhou University. Written informed consent was obtained from all participants before conducting the study.

### Semen collection

We used semen parameters of their first ejaculate for statistical analysis. Semen samples were collected by masturbation into wide-mouth containers, in a private room, after 2–7 days of sexual abstinence. All samples were incubated at 37 °C until liquefaction and analyzed within 1 h.

### Semen analysis

Semen analyses were performed following the WHO guidelines^20,21^. The following parameters were measured: volume, sperm concentration, total sperm count, progressive motility (grades a + b), and total motility (grades a + b + c). Assuming that the density of semen is 1 g/ml^2^, semen volume was estimated by weighting. Sperm concentration was counted and motility was assessed by the conventional method using the Makler counting chamber (Sefi Medical Instruments, Haifa, Israel). The Makler counting chamber was prewarmed at 37 °C prior to use^5^. A 10μL drop of well-mixed semen was placed onto a warmed Makler counting chamber and covered gently with the cover glass, then examined at a total magnification of × 400. Sperm count was performed in 10 squares of the chamber and recorded by the cytometer^5^. Motility was assessed in at least 100 sperms and expressed as a percent of motile sperm^8^.

### Quality control

To reduce variation in the assessment of semen quality, all assessments were repeated in duplicate. Independent duplicate counts in the two sides of the Marler counting chamber were compared. The procedure was repeated when the difference between the two counts was above 10%. During the entire period of the study, all the laboratory technicians have received the same training. Internal quality control (IQC) consisted of monthly assessment of technicians by the director of the laboratory. All semen analyses were conducted by the same two well-trained technicians. In the measurement of sperm concentrations, intra-technician coefficients of variation for the two technicians were 1.3 and 2.1 percent; the inter-technician coefficient of variation was 3.1 percent. In the assessment of motility, intra-technician coefficients of variation for the two technicians were 2.3 and 3.1 percent; the inter-technician coefficient of variation was 5.1 percent. The technicians, director of the laboratory, and analytical methods did not change during the entire period of the study. Our laboratory participated in the external quality assurance (EQA) scheme organized by the Guangdong Province Human Sperm Bank, and the results did not show any temporal trend in the assessment level of our laboratory. IQC and EQA were conducted during the entire period of the study.

### Statistical analyses

SPSS (ver. 20.0) software (SPSS Inc., Chicago, Illinois, USA) was used for statistical analysis. To evaluate the normal distribution of the data, the Kolmogorov–Smirnov (K–S) test was used. Normally distributed continuous data were presented as mean ± standard deviation (SD) and not normally distributed continuous data were presented as median with interquartile range. Non-transformed semen parameters were used in the linear regression analyses because the sample size was large enough to provide reliable results without any transformation. Linear regression analysis was used to assess rates of increase per year in age, BMI, and sexual abstinence duration. Linear regression analysis was also used to examine trends over time in main semen parameters. After adjustment for age, BMI, and sexual abstinence duration, we evaluated changes in main semen parameters over time using multiple linear regression analyses Statistical significance was set at *P* < 0.05.

## Results

**Figure 1:**
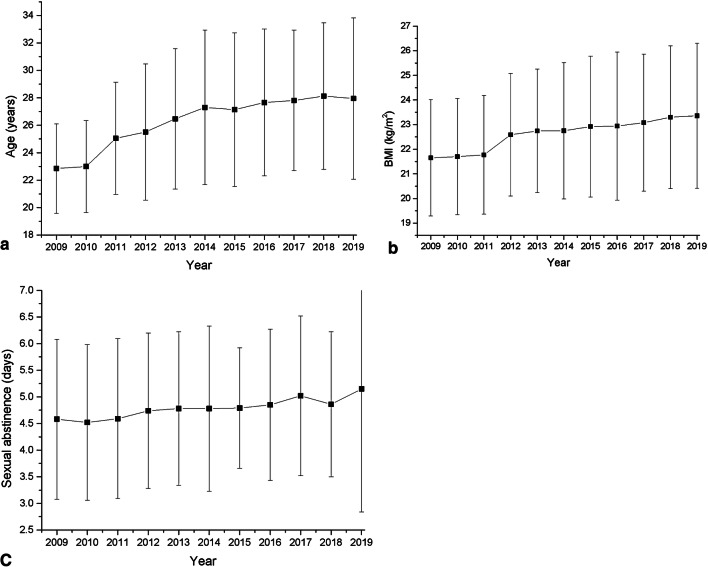
Changes in age (**a**), BMI (**b**), and days of sexual abstinence (**c**) between 2009 and 2019. Values are expressed as mean ± standard deviation (SD).

**Figure 2:**
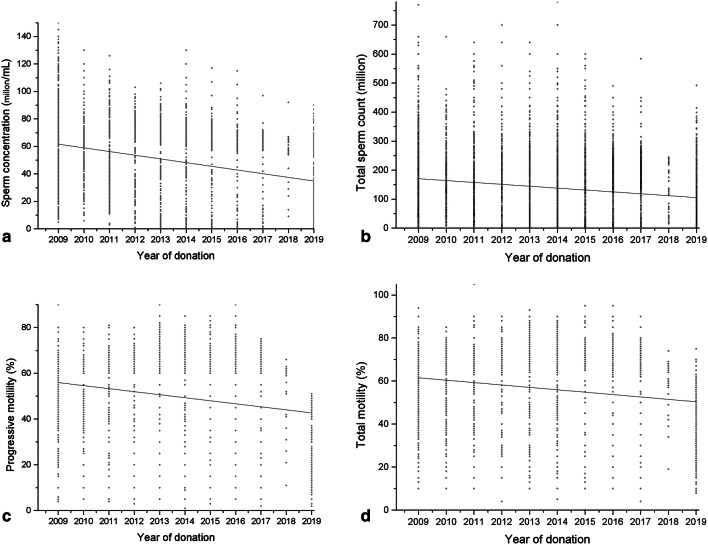
Multiple regression analysis of the change in main semen parameters over time. After adjustment for age, BMI, and days of sexual abstinence, significant decreases in sperm concentration (**a**), total sperm count (**b**), progressive motility (**c**), and total motility (**d**) were observed.

As shown in Table 1 and Fig. 1, the average age increased from 22.85 ± 3.26 years in 2009 to 27.88 ± 5.79 years in 2019, with an average annual rate of 1.6%. The average BMI increased from 21.65 ± 2.36 kg/m^2^ in 2009 to 23.36 ± 2.94 kg/m^2^ in 2019, with an average annual rate of 0.7%. The average days of sexual abstinence increased from 4.58 ± 1.50 days in 2009 to 5.15 ± 2.31 days in 2019, with an average annual rate of 1.1%. As shown in Tables 1 and 2, the number of sperm donor candidates increased gradually, reached its peak in 2014, and then decreased gradually. As presented in Tables 2, 3, and Fig. 2, the sperm concentration decreased from 62.0 million/mL in 2009 to 32.0 million/mL in 2019 (*P* < 0.001), with an average annual rate of 3.9%. The total sperm count decreased from 160.0 million in 2009 to 80.0 million in 2019 (*P* < 0.001), with an average annual rate of 4.2%. The progressive motility decreased from 54.0% in 2009 to 40.0% in 2019 (*P* < 0.001), with an average annual rate of 2.5%. The total motility decreased from 60.0% in 2009 to 46.0% in 2019 (*P* < 0.001), with an average annual rate of 1.9%.

**Table 1:** General characteristics of sperm donors.

**Year**	***n***	**Age (years)**	**BMI (kg/m**^**2**^**)**	**Sexual abstinence (days)**
2009	907	22.85 ± 3.26	21.65 ± 2.36	4.58 ± 1.50
2010	408	22.99 ± 3.35	21.70 ± 2.36	4.52 ± 1.46
2011	981	25.05 ± 4.09	21.77 ± 2.41	4.59 ± 1.50
2012	751	25.51 ± 4.97	22.59 ± 2.49	4.74 ± 1.46
2013	3,258	26.47 ± 5.12	22.74 ± 2.51	4.78 ± 1.44
2014	5,446	27.30 ± 5.63	22.75 ± 2.77	4.78 ± 1.55
2015	3,759	27.14 ± 5.59	22.92 ± 2.86	4.79 ± 1.13
2016	2,523	27.66 ± 5.35	22.94 ± 3.01	4.85 ± 1.42
2017	2,343	27.81 ± 5.12	23.08 ± 2.78	5.02 ± 1.50
2018	1,412	28.13 ± 5.34	23.30 ± 2.90	4.86 ± 1.36
2019	2,148	27.88 ± 5.79	23.36 ± 2.94	5.15 ± 2.31
Rates of increase per year		1.6%	0.7%	1.1%

Data are expressed as mean ± standard deviation (*n* = number of sperm donor candidates).

**Table 2:** Descriptive characteristics of participants’ semen parameters between 2009 and 2019.

**Year**	***n***	**Sperm concentration (million/mL)**	**Total sperm count (million)**	**Progressive motility (%)**	**Total motility (%)**
		**Median [IQR 25–75]**	**Median [IQR 25–75]**	**Median [IQR 25–75]**	**Median [IQR 25–75]**
2009	907	62.0 [40.0–77.0]	160.0 [100.0–225.0]	54.0 [40.0–64.0]	60.0 [48.0–69.0]
2010	408	60.0 [40.0–74.0]	157.0 [104.5–219.0]	60.0 [45.25–65.0]	65.0 [50.0–70.0]
2011	981	56.0 [39.0–73.0]	150.0 [80.0–210.0]	50.0 [40.0–65.0]	60.0 [45.0–70.0]
2012	751	53.0 [30.0–72.0]	138.0 [80.0–195.0]	50.0 [40.0–65.0]	60.0 [45.0–70.0]
2013	3,258	51.0 [31.0–68.0]	126.0 [75.0–200.0]	50.0 [35.0–65.0]	60.0 [45.0–70.0]
2014	5,446	50.0 [30.0–65.0]	120.0 [70.00–190.0]	50.0 [40.0–60.0]	60.0 [45.0–70.0]
2015	3,759	47.0 [27.0–62.0]	118.0 [63.0–183.0]	50.0 [40.0–62.0]	60.0 [45.0–70.0]
2016	2,523	45.0 [25.0–60.0]	111.0 [61.0–171.1]	49.0 [39.0–59.0]	59.0 [49.0–69.0]
2017	2,343	42.0 [22.0–57.0]	100.0 [60.0–160.0]	48.0 [38.0–58.0]	57.0 [42.0–64.0]
2018	1,412	34.0 [24.0–55.0]	80.0 [50.0–166.0]	41.0 [31.0–51.0]	54.0 [40.0–60.0]
2019	2,148	32.0 [12.00–54.0]	80.0 [25.0–135.0]	40.0 [21.0–43.0]	46.0 [30.0–49.0]
Rates of decrease per year		3.9	4.2	2.5	1.9

Rates of decrease per year were assessed using multiple regression analysis adjusting for age, BMI, and days of sexual abstinence.

*IQR* interquartile range.

**Table 3:** Linear regression analyses of temporal trend in semen parameters before and after adjustment for age, body mass index (BMI), and sexual abstinence duration.

**Semen parameter**	**Unadjusted**			**Adjusted**		
	***β***	**95% CI**	***P value***	***β***	**95% CI**	***P value***
Sperm concentration	− 2.53	− 2.44 to − 2.62	< 0.001	− 2.36	− 2.28 to − 2.45	< 0.001
Total sperm count	− 7.57	− 7.13 to − 8.00	< 0.001	− 7.30	− 6.87 to − 7.74	< 0.001
Progressive motility	− 1.49	− 1.41 to − 1.56	< 0.001	− 1.32	− 1.25 to − 1.40	< 0.001
Total motility	− 1.24	− 1.17 to − 1.32	< 0.001	− 1.12	− 1.05 to − 1.19	< 0.001

*CI* confidence interval.

## Discussion

Semen analysis is the most important and the most widely used diagnostic methods to assess male fertility potential, and it plays a critical role in the field of andrology. Although alternate tests have been developed based on more functional aspects (such as acrosome reaction, capacitation, and sperm penetration), semen analysis is still the primary method to assess male fertility potential^22^. In this retrospective study, we included 23,936 sperm donor candidates who were recruited by the Henan Human Sperm Bank of China between 2009 and 2019. As far as we know, this is the first study on time trends in semen quality among Chinese men in Henan Province.

Studies^2–8,23–25^ suggest that semen quality is declining globally, however, the debate remains open due to the possible effects of ethnic and geographical differences. In contrast, several other studies have reported no significant change in semen quality^9–12^. The sperm concentration decreased from 62.0 million/mL in 2009 to 32.0 million/mL in 2019, with an annualized rate of 3.9% in this study. The annualized rate of decline in the sperm concentration was lower than that reported in Shandong (6.89%)^6^ and America (4% per year)^23^, but it was higher than that reported in Paris (2.1% per year)^2^ and Marseille (1.5% per year)^4^. The total sperm count decreased from 160.0 million in 2009 to 80.0 million in 2019, with an average annual rate of 4.20% in this study. The annualized rate of decline in the total sperm count was lower than that reported in Shandong (9.84% per year)^6^, but it was higher than that reported in Marseille (1.6% per year)^4^ and America (3% per year)^23^. The progressive motility decreased from 54.0% in 2009 to 40.0% in 2019, with an average annual rate of 2.5% in this study. The annualized rate of decline in the progressive motility was lower than that reported in Marseille (5.5% per year)^4^, but it was higher than that reported in Shandong (1.37% per year)^6^. The total motility decreased from 60.0% in 2009 to 46.0% in 2019, with an average annual rate of 1.9% in this study. The annualized rate of decline in the total motility was lower than that reported in America (2% per year)^23^, but it was higher than that reported in Paris (0.6% per year)^2^ and Marseille (0.4% per year)^4^. The possible reasons for the discrepancy are as follows: (1) small sample size are used in the previous study^10,11^; (2) subjects in these studies are fertile men^2,3,11^ or infertile men^5,21,25^; (3) there are differences in lifestyle habits, ethnicity, and geographical regions^2–11,22–25^; (4) the different study periods^2–11,22–25^.

Our results clearly illustrate that semen quality is declining during the past 11 years in Henan Province, China. Our findings support previous reports that semen quality is declining in other parts of the world^2–8^. The possible etiologies of the declining semen quality among Chinese men are unclear. It has been suggested that the increased incidence of male reproductive abnormalities reflects adverse effects of environmental and lifestyle factors, such as environmental exposure to pollutants and toxicants, obesity, dietary patterns, smoking, alcohol intake, and stress^13,14,26–30^.

A strength of this study was the study population and study design. All the sperm donor candidates in our study were not selected for marital status or fertility in order to reduce selection bias. Since the opening of the Henan Human Sperm Bank, the strictest criteria have been applied. Semen analysis was performed following the WHO guidelines^20,21^. All semen analyses were conducted by the same two well-trained technicians. To minimize measurement bias, the same instruments used in this study were the same and technicians adhered to strict quality control during the entire period of the study. This is a single-center study, which avoids between-center variability in the evaluation of semen analysis. A previous study has indicated that a single semen sample is fairly representative of the overall semen quality of healthy men in China^31^. In this study, we included only the first ejaculate provided by each sperm donor candidate. Ethnic and geographical variations should be taken into account when investigating whether semen quality has changed over time. To reduce ethnic and geographical variations, all participants in this study were randomly selected from the Chinese Han population in Henan Province, China.

One limitation of this study is the lack of the factors affecting semen quality such as smoking, stress, food habits, alcohol abuse, and occupation. The population of this study represents only one geographical region of China. And our study focused on a limited age range (20–44 years). Another limitation of this study is that our findings might not be adequately representative of the whole Chinese population.

Our results indicated that semen quality among sperm donor candidates had decreased during the study period in Henan Province, China. This study is important because it has a relatively large sample size and long study duration, which would have strong statistical power.

## Data Availability

The datasets used during the current study are available from the corresponding author on a reasonable request.
